# Supplementary material for: Probing the Nano-Assembly Leading to Periodic Gratings in Poly(p-dioxanone)
Source: Nanomaterials (Basel). 2023 Sep 28;13(19):2665. doi: 10.3390/nano13192665 (PMC10574605; doi:10.3390/nano13192665)
Supplement: Supplementary file 1 [file nanomaterials-13-02665-s001.zip › nanomaterials-2608668-supplementary.pdf]

# Supporting Information

# Probing the Nano-Assembly Leading to Periodic Gratings in Poly(p-dioxanone)

*Min-Han Hao*[1], Selvaraj Nagarajan[1]*, *and Eamor M. Woo*[1]*

[1]Department of Chemical Engineering, National Cheng Kung University

No. 1, University Road, Tainan, 701-01, Taiwan

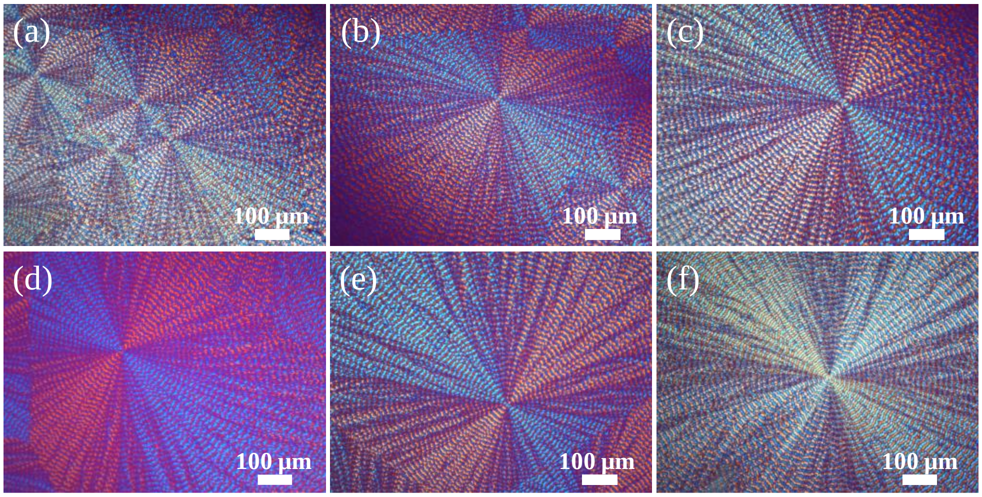


**Figure S1.** POM graphs of PPDO/PVA (95/5) blend crystallized with top-glass cover at various $T_c$s (a) 45 $^{o}$C, (b) 50 $^{o}$C, (c) 55 $^{o}$C, (d) 60 $^{o}$C (e) 65 $^{o}$C, (f) 70 $^{o}$C.

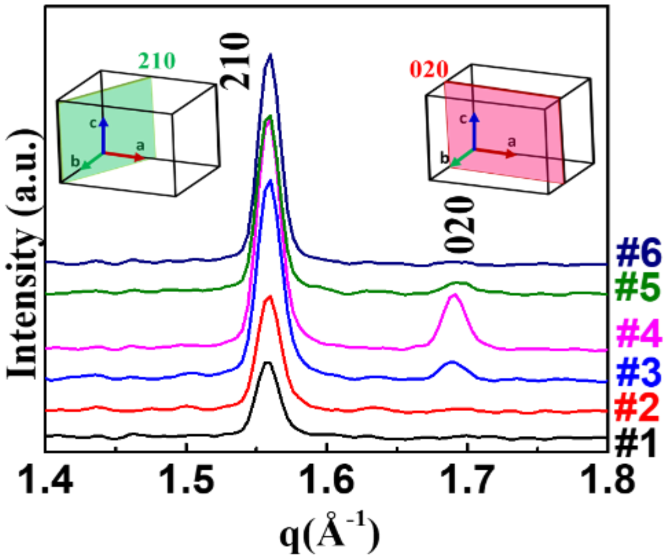


**Figure S2**: 1D-WAXD profiles of spot #1 to #6 in a ring-banded spherulite of PPDO/PVA (95/5) blend crystallized at $T_C$= 65 °C.

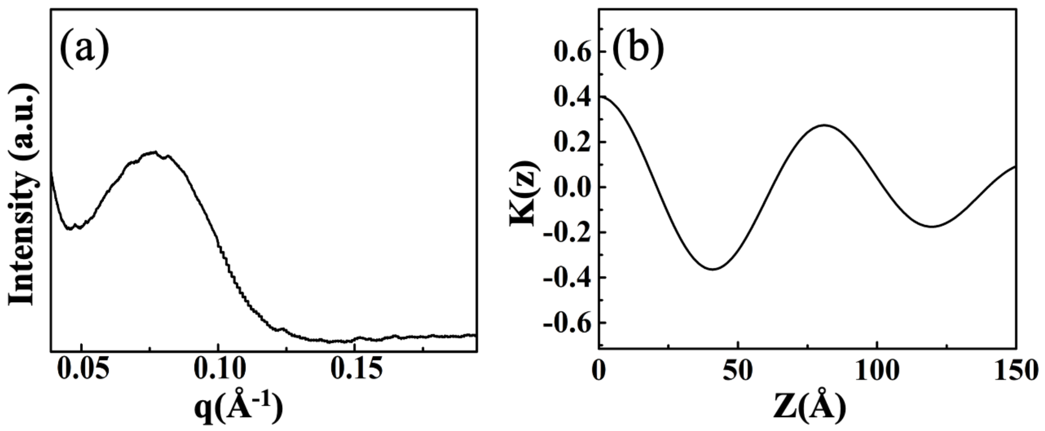


**Figure S3.** Synchrotron microbeam X-ray diffraction results (a) 1D correlation profile, and (b) 1D correlation function of PPDO/PVA (95/5) ring-banded spherulite crystallized at $T_C$ = 65 ºC.
